# Baltic Sea coastal sediment-bound eukaryotes have increased year-round activities under predicted climate change related warming

**DOI:** 10.3389/fmicb.2024.1369102

**Published:** 2024-03-26

**Authors:** Songjun Li, Emelie Nilsson, Laura Seidel, Marcelo Ketzer, Anders Forsman, Mark Dopson, Samuel Hylander

**Affiliations:** ^1^Centre for Ecology and Evolution in Microbial Model Systems, Linnaeus University, Kalmar, Sweden; ^2^Department of Ecology, Environment and Plant Sciences, Stockholm University, Stockholm, Sweden; ^3^Department of Biology and Environmental Sciences, Linnaeus University, Kalmar, Sweden

**Keywords:** marine, RNA transcripts, diversity, community structure, functional activity

## Abstract

Climate change related warming is a serious environmental problem attributed to anthropogenic activities, causing ocean water temperatures to rise in the coastal marine ecosystem since the last century. This particularly affects benthic microbial communities, which are crucial for biogeochemical cycles. While bacterial communities have received considerable scientific attention, the benthic eukaryotic community response to climate change remains relatively overlooked. In this study, sediments were sampled from a heated (average 5°C increase over the whole year for over 50 years) and a control (contemporary conditions) Baltic Sea bay during four different seasons across a year. RNA transcript counts were then used to investigate eukaryotic community changes under long-term warming. The composition of active species in the heated and control bay sediment eukaryotic communities differed, which was mainly attributed to salinity and temperature. The family level RNA transcript alpha diversity in the heated bay was higher during May but lower in November, compared with the control bay, suggesting altered seasonal activity patterns and dynamics. In addition, structures of the active eukaryotic communities varied between the two bays during the same season. Hence, this study revealed that long-term warming can change seasonality in eukaryotic diversity patterns. Relative abundances and transcript expression comparisons between bays suggested that some taxa that now have lower mRNA transcripts numbers could be favored by future warming. Furthermore, long-term warming can lead to a more active metabolism in these communities throughout the year, such as higher transcript numbers associated with diatom energy production and protein synthesis in the heated bay during winter. In all, these data can help predict how future global warming will affect the ecology and metabolism of eukaryotic community in coastal sediments.

## Introduction

Climate change is a world-wide environmental problem caused by anthropogenic greenhouse gas emissions that results in an increase in average surface temperature (IPCC et al., 2008). As the largest ecosystem on Earth with an important role in geochemical cycling of key elements such as Fe, S, and Mn, the marine ecosystem is influenced by climate change with associated effects such as an increased ocean surface temperature of 1°C in the last century (IPCC et al., 2008; Abraham et al., 2013; Garcia-Soto et al., 2021) and higher dissolved carbon dioxide (CO_2_) concentrations (Connell et al., 2013). This leads to further problems such as acidification, salinity changes, water stratification, deoxygenation, and sea level rise (Connell et al., 2013; Breitburg et al., 2018; Malhi et al., 2020). In addition, mounting evidence indicates that climate change alters the biodiversity and community compositions of marine ecosystems. These include tropical regions experiencing species loss while temperate regions might experience increasing diversity as species migrate to the poles (Alabia et al., 2020) that has potentially far reaching implications for ecosystem services (Staniczenko et al., 2017).

Despite only consisting of 4% of the earth’s total area and 11% of the world’s oceans, coastal zones contain more than a third of the world’s human population and contribute with 90% of the catch from marine fisheries (Barbier, 2017). Coastal areas also provide many additional ecosystem services including carbon storage in estuaries and sediments, contaminant removal, and storm plus flooding buffering (Canuel et al., 2012). The coastal areas are also responsible for the majority of atmospheric methane emission from the marine environment (Borges et al., 2016; Weber et al., 2019). Eukaryotes are abundant in coastal systems and play important ecological roles by serving as both primary producers and consumers (Nagarkar et al., 2018). For example, they graze on prokaryotes (Massana et al., 2004) and deliver energy to higher trophic levels through the food web. Moreover, eukaryotes are also involved in the benthic–pelagic exchange process between the sediment and open water (Marcus and Boero, 1998) with the transfer of individual organisms as well as elements such as phosphorous and nitrogen (Fanning et al., 1982). One important habitat for eukaryotes is the benthic sediment, although it has received less attention compared to the pelagic environment (Salonen et al., 2019). The eukaryotic communities in sediments are complex and diverse, including various benthic macrofauna (e.g., Bivalvia), meiofauna (e.g., nematodes, protists), and algae (e.g., diatoms) (Bik et al., 2012; Broman et al., 2019b). Moreover, sediment acts as a reservoir for resting stages of phytoplankton and zooplankton (Brendonck and De Meester, 2003; Suikkanen et al., 2010) and harbors larvae of semi-aquatic insects for some time before they emerge to the terrestrial phase (Mason et al., 2022). The coastal biodiversity is threated by climate change and anthropogenic activities (Holon et al., 2018), resulting in many species disappearing (Pan et al., 2013), and such that it may correspondingly affect ecosystem processes. For example, low sediment biodiversity can decrease the coastal ecosystem’s stability and resistance, making it vulnerable to invasive species or other forms of disturbance (Levin et al., 2001). Therefore, it is essential to better understand how the structure and activity of coastal benthic eukaryotic communities is influenced by global warming.

The Baltic Sea is one of the largest brackish water areas in the world that it is relatively isolated due to a narrow connection to the North Sea (Stigebrandt, 2001). This sea has suffered from a high level of eutrophication over an extended time due to anthropogenic nutrient loading and atmospheric deposition of primarily nitrogen and phosphorus (Knuuttila et al., 2011). This eutrophication contributes to increased biomass production and elevated oxygen consumption (Reusch et al., 2018). In the last century, the area of year-round hypoxic “dead zones” in the Baltic Sea has expanded ten-fold (Carstensen et al., 2014). Correspondingly, the dead zone eukaryotic community structure has also been modified with an increased abundance of species tolerating low oxygen conditions such as nematodes (Broman et al., 2020) and lower hatching of zooplankton resting stages (Broman et al., 2015). While higher global warming-related oxygen consumption (Schmidtko et al., 2017) will likely magnify the influence of eutrophication and algae blooms (Reusch et al., 2018), it is not well known how the benthic eukaryotic community and especially the active groups will respond.

This research was conducted in a Baltic Sea bay that has been used as a discharge recipient of warm water from a nuclear power plant for more than 50 years, which has raised the average water temperature by approximately 5.1°C above a nearby, unaffected, control bay. This temperature difference is within the same order of magnitude of the expected temperature increase for the Baltic Sea (Andersson et al., 2015). Therefore, this bay can be regarded as a natural laboratory to study the influence of long-term climate change in a Baltic Sea coastal ecosystem (Seidel et al., 2022a). Previous studies of this model system have uncovered that the sediment prokaryote communities in the heated versus the control bay show a weakened resilience with microbial RNA transcripts for stress in the heated bay despite exposure to > 50 years of warming (Seidel et al., 2022a). In addition, the sediment surface microbial community (< 2 cm of depth) in the warmed conditions exhibited a higher diversity due to shallowing of geochemical layers (Seidel et al., 2022b).

In this study, a metatranscriptomic dataset from the heated and control Baltic Sea bay sediments at four seasonal time points over a year was interrogated to investigate whether and how the eukaryotic community composition and their active functions differed between the two bays and change over time. It was hypothesized that heating of coastal marine system leads to a difference in the species composition of sediment-bound eukaryotic communities as well as different seasonal dynamics in their transcriptome signatures, such as overall increased activities in the winter.

## Materials and methods

### Sampling sites and sediment cores

**FIGURE 1:**
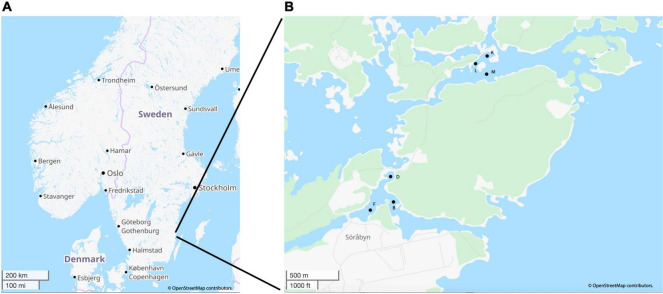
Map of the general sampling are near Oskarshamn, Sweden **(A)** and a zoomed in image of the heated and control bays with specific sampling points **(B)**. Image generated and adapted in OpenStreetMap, licensed under CC BY-SA 2.0.

The sediment sample cores were collected using a kajak gravity corer from three sites in each of the heated (B: N 057° 25.259′ E 016° 40.130′, D: N 057° 25.387′ E 016° 40.104′, and F: N 057° 25.220′ E 016° 39.895′) and control (K: N 057° 26.011′ E 016° 41.022′, L: N 057° 25.964′ E 016° 40.914′, and M: N 057° 25.907′ E 016° 40.992′) Baltic Sea bays (Figure 1). The water depths for the sites were: B- 3 m, D- 1.2 m, F- 2.4 m, K- 2.8 m, L- 1.6 m, M- 4.9 m. Samples were collected in May, June, and November 2018 plus March in 2019 as previously described (Broman et al., 2019a; Seidel et al., 2022b). Briefly, sediments were collected with a 50 cm-long kayak-type gravity corer, immediately sliced in the field, and the 0–1 cm sediment surface was aseptically retained for nucleic acid extractions along with chemical analyses (Broman et al., 2019a). All nucleic acid samples were flash frozen in liquid nitrogen before being returned to the laboratory on the same day and stored in a −80°C freezer.

### Temperature measurements and physiochemical analyses

Surface water temperature (1 m below the sea surface) was monitored in the two bays by using data loggers (HOBOware, Onset Computer Corporation, USA) at each of the sampling sites from December 2017 until November 2019. In addition, the bottom water temperature was measured *in situ* (Multiline™ sensor, WTW™) during each sampling time point (Seidel et al., 2022b). The chemical analyses of pore water including oxygen, pH, salinity, organic matter, nitrate, nitrite, ferrous iron, total iron, and sulfate on sediment were conducted as previously described (Broman et al., 2019a).

### RNA extractions and sequencing

RNA was extracted from the sediment 0–1 cm depth fractions using the RNeasy^®^ PowerSoil Total RNA Kit (QIAGEN) and the phenol/chloroform/isoamyl alcohol method as previously described (Seidel et al., 2022b). Total RNA samples were sent to the DOE Joint Genome Institute (JGI) at the Lawrence Berkeley National Laboratory, Berkeley, USA where they performed sequencing on the Illumina NovaSeq600 platform to produce sequences with 2 × 151 bp read length. Quality control was conducted to eliminate contaminants and ribosomal RNA reads by using BBDuk (v. 38.75) and BBMap (Bushnell, 2023) that resulted in an average of 64.83 % of the reads being retained [as previously reported (Seidel et al., 2022b)].

### Bioinformatics and statistical analyses

The filtered mRNA reads provided by JGI were co-assembled by Megahit v.1.2.9 (Li et al., 2015) with default settings. TransDecoder v.5.5.0 (Haas, 2023) with default settings (first LongOrfs function then Predict function) was used to identify candidate coding regions within the transcript sequences, which generated open reading frames (ORFs) from the assembled contigs. After that, Bowtie2 with default settings v.2.3.5.1 (Langmead and Salzberg, 2012) was used align the sequencing reads. The Bowtie2 output was used to generate a counts table using FeatureCounts v.2.0.3 (Liao et al., 2014) with standard settings. Taxonomic annotation was performed using the software Eukulele v.2.0.2 (Krinos et al., 2023) in default settings against the reference PhyloDB v.1.075 database (Allen, 2020). Since the focus of this paper was on eukaryotes, only contigs annotated as within the Eukaryote domain were retained for further analyses. Functional annotation used the automatic annotation servers GhostKOALA in KEGG (Kyoto Encyclopedia of Genes and Genomes) website, which is an internal annotation tool for KEGG Orthology assignment (Kanehisa et al., 2016).

The Alpha Diversity indices (Shannon’s H and evenness) were normalized by scaling with ranked subsampling in package “SRS” (v.0.2.3), and then calculated by the package “vegan” (v.2.6-4) (Oksanen, 2010) in R (v.4.3.1). A linear regression model was used to test for significant differences between the bays and among the different sampling months. The model was created by the “stats” package (v.4.1.2) (R Core Team, 2018) and followed by additional pairwise comparison of bays on each sampling month using the package “emmeans” (v.1.8.5) (Lenth, 2023) in R. PerMANOVA with 999 permutations was used to test for statistical differences on eukaryotic RNA transcript community comparing both bays, using the “adonis()” function from the package “vegan” (v.2.6-4) (Oksanen, 2010) and the canonical correspondence analysis was also done by package “vegan” (v.2.6-4) (Oksanen, 2010) with “cca()” function. The variance inflation factor (VIF) based on the canonical correspondence analysis from the “vegan” package was used to determine if the measured environmental variables added new information to the differences between the two groups. Permutations ANOVA (*n* = 999 permutations) was used for testing the statistical significance of each environmental variable. Package “ANCOM-BC2” (v.3.16) (Lin and Peddada, 2020) was used for differential RNA transcript abundance analysis at phylum level between the bays in each sampling month.

Differential RNA transcript expression analysis was performed using the “edgeR” package (v. 3.36.0) in R (Robinson et al., 2010). The two bays (*n* = 12 per bay) were first modeled as contrast factors to give differentially expressed genes over the year. Then both bays and months were modeled together as contrast factors, with the different sampling sites as replicates (*n* = 3 per bay per month) to give differentially expressed genes among each month. To ensure the downstream statistical analysis would not be affected by low counts, a cutoff (> 113 counts and at least in three sample libraries) was set to filter out low count transcripts. The choice for this cutoff was based on 10/L where L was the minimum library size (88795 RNA transcripts) and at least for three libraries as each bay-month group contained three replicates (Chen et al., 2016). Then the differential expression analysis relative to fold change threshold was performed by “glmTreat()” function inside the edgeR package. This function is analogous to the TREAT method for microarrays and modifies the statistical test to detect expression changes greater than a specified threshold. The threshold used in this study was log2(1.5) as a standard value (Chen et al., 2016) to remove transcripts with fold changes below this threshold. The Benjamini–Hochberg method was used as *p*-adjustment type and the *p*-value setting was 0.05.

## Results

### RNA sequencing

The RNA sequencing generated a total of 1,437,330,588 reads in 24 sediment core samples (min. 28,035,392 and max. 68,172,798 reads) giving on average 64.83% mRNA reads after rRNA filtering. Assembly of the filtered RNA reads generated 4,214,024 contigs with 55.47% (2,337,566) assigned a taxonomy of which 12.72% (297,242) belonged to the Eukaryota (Supplementary Figure 1 and Supplementary Table 1). A rarefaction curve evaluation was done on the filtered eukaryotic RNA reads (Supplementary Figure 5).

### RNA transcript based eukaryotic diversity

**FIGURE 2:**
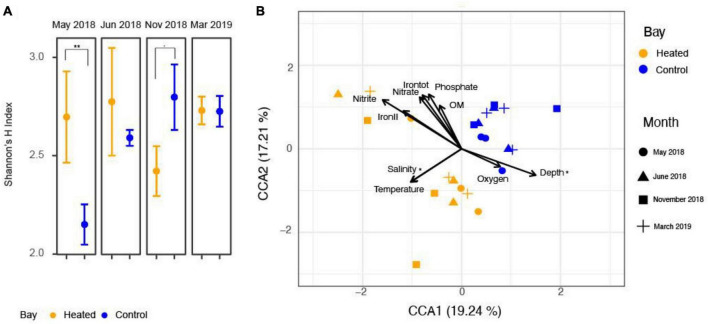
Shannon ìs H index in family level **(A)** along with canonical correspondence analysis based on mRNA transcripts with relevant physiochemical parameters **(B)** for the heated (orange) and control (blue) bays for each sampling month. The asterisk denotes statistically significant (*p* < 0.05), and the dot denotes a *p*-value between 0.05 and 0.1.

A canonical correspondence analysis of RNA transcript-based eukaryote beta diversity (Figure 2B) showed significantly different eukaryotic RNA transcript community compositions between the two bays (PERMANOVA, df = 1, *F* = 3.85, *p* = 0.001). Among the physiochemical parameters separating the eukaryotic communities, salinity and water depth were two significant variables from the permutation ANOVA test (Supplementary Tables 3, 4). Salinity and bottom water temperature best fitted the separation between the heated and control bay while water depth, bottom water oxygen concentrations, and other geochemical parameters contributed more to the site variation, especially site D in the heated bay (left top part in Figure 2B).

Seasonal patterns of eukaryotic community Shannon’s H index were observed between the two bays at the family level (ANOVA: Bay, df = 1, *F* = 0.84, *p* = 0.37; Month, df = 3, *F* = 1.87, *p* = 0.18; Bay-month interaction, df = 3, *F* = 3.84, *p* = 0.03; Figure 2A). Therefore, single effects were insignificant but there was an interactive effect of month and bay on Shannon’s H index. More specifically in May, the heated bay had a significantly higher Shannon’s H index diversity compared to the control bay (pairwise comparison, 2.80 ± 0.3 and 1.96 ± 0.3, *p* = 0.01) while the control bay had higher diversity in November (pairwise comparison, 2.96 ± 0.3 and 2.38 ± 0.3, *p* = 0.08). In June and March, there were no statistical differences between the heated and control bays (pairwise comparison, June: 2.92 ± 0.3 and 2.64 ± 0.2, *p* = 0.37, March: 2.85 ± 0.3 and 2.85 ± 0.3, *p* = 0.98). The Shannon’s evenness followed the same pattern as for the H index (Supplementary Figure 2).

### Community composition on phylum and family level

**FIGURE 3:**
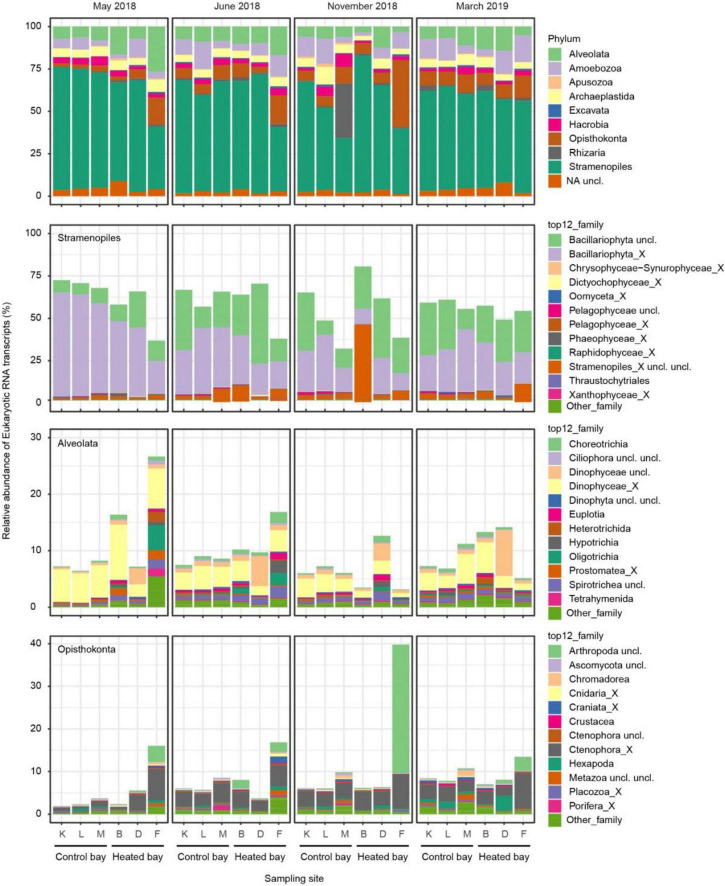
Relative abundance of eukaryotic mRNA transcripts at the level of phylum in the control and heated bays over the four sampling months (top). The Stramenopiles, Alevolata, and Opisthokonta phyla were further split into their respective relative abundance of families.

The most abundant RNA transcript-based phylum was the diverse and often single-celled Stramenopiles (58% in total; Figure 3) encompassing both photosynthetic and non-photosynthetic members (Dorrell et al., 2017) that consisted of > 50% average relative abundance of all samples in both the heated plus control bays and all sampling months. The dominant family within the Stramenopiles was Bacillariophyta (combined unclassified Bacillariophyta and Bacillariophyta_X, i.e., diatoms), especially in the control bay during spring-summer (May and June). Alveolata was the second most abundant RNA transcript-based phylum (10%). However, the Alveolata displayed a different pattern with a tendency to higher relative abundance in the heated bay, especially during spring-summer (May and June). The dominant family within the Alveolata was the dinoflagellates Dinophyceae (combined unclassified Dinophyceae and Dinophyceae_X). Alveolata was followed in dominance by the Opisthokonta phylum (8%) that lacked a clear pattern between the two bays or among seasons but had a high relative abundance in site F in the heated bay compared to the other sites (especially in November with the Arthropoda family). The dominant families within the Opisthokonta were the aforementioned Arthropoda along with the marine gelatinous animals Ctenophora (Ctenophora unclassified and Ctenophora_X). However, the disparities in the community composition between the bays in each sampling month were small based on differential abundance analysis, and only the phyla Stramenopiles, Opisthokonta, and some rare taxa were statistically different in March (Supplementary Table 2).

### mRNA transcript based activity

After filtering out genes with insufficient counts for statistical analysis, eukaryotic RNA transcripts encoding 1,319 genes had a differential count between the two bays (lfc = log_2_ 1.5, *p* < 0.05). In total for all sampling occasions, 147 genes had significantly higher transcript counts in the control bay compared to the heated bay and 213 were higher in the heated bay (Supplementary Figure 3). Disregarding effects of sampling month, common taxa such as Pelagophyceae, Bacillariophyta, and Dinophyta had significantly different RNA transcript counts in both bays that encoded diverse functions in cell metabolism. Many of the taxa displaying differential transcript counts were diatoms, dinoflagellates, and other algae that are typically primary producers containing chlorophyll with a pelagic or benthic lifestyle [e.g., in biofilms or as resting stages with some mRNA to maintain viability (Coyne and Craig Cary, 2005)]. Among the less common taxa, there were some bay-specific genes with differential transcript counts. For example, Spirotrichea (e.g., ciliates), the Ctenophora (gelatinous zooplankton with benthic stage), and Archaeplastida (Mamiellophyceae/Zygnemophyceae/Trebouxiophyceae) only had significantly more transcript counts in the heated bay. In contrast, a few Euglenozoa RNA transcripts were only significantly increased in the control bay.

### Month-specific RNA transcript differences

**FIGURE 4:**
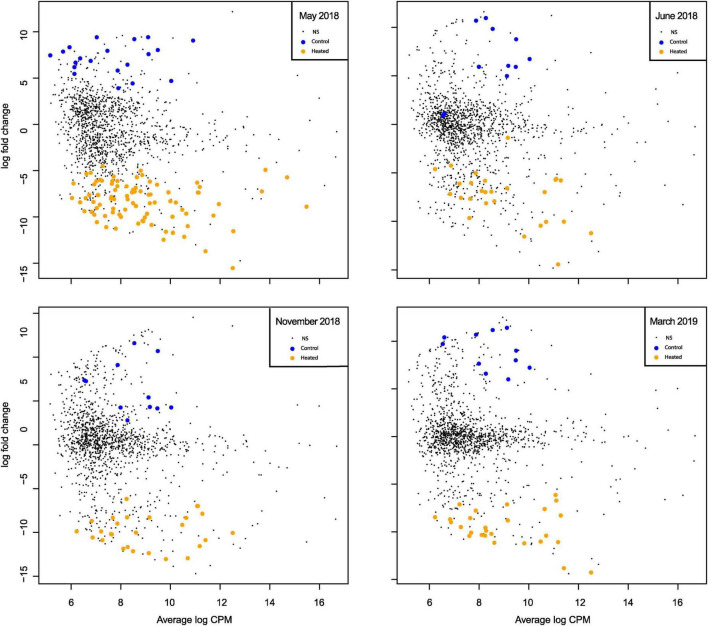
Mean-difference plot of transcript counts per million (CPM) between heated bay and control bay in the separate months. Genes with fold-changes significantly greater than 1.5 (*p* < 0.05) are highlighted for the heated (orange) and control (blue) bays for each sampling month. NS signifies genes with no significant difference between the two bays.

The seasonal dynamics in significantly different RNA transcripts [lfc = log2(1.5), *p* = 0.05] was studied by considering sampling months separately (Figure 4). There were overall more significant increased transcript numbers in May (*n* = 87) in the heated bay compared to the control bay (*n* = 20; Figure 4), which was in line with the patterns in the community diversity (Figure 2A) whereby the alpha diversity was higher in the heated bay at that time point.

**FIGURE 5:**
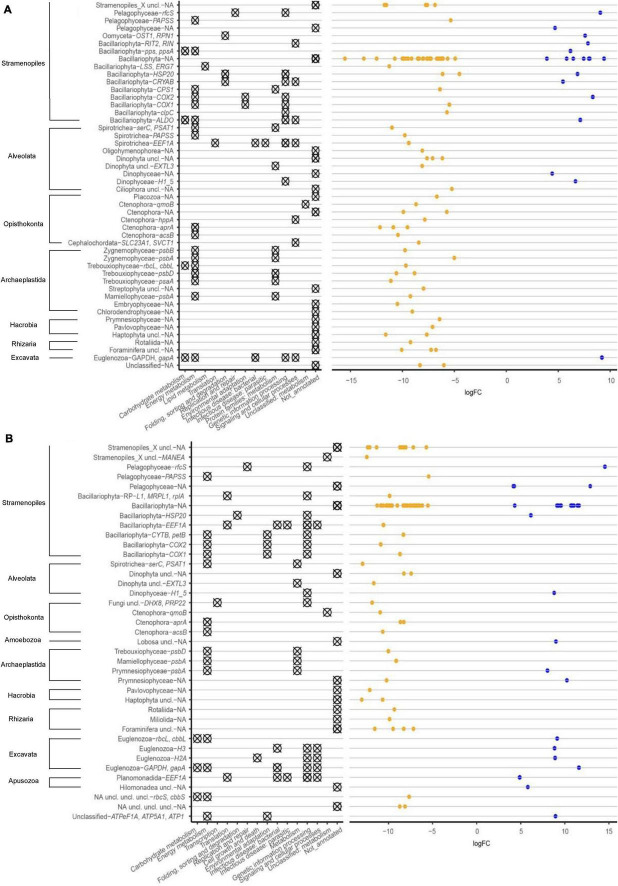
Taxonomy and metabolic functional groups of differential RNA transcript numbers (logFC) for genes between heated bay and control bay in May **(A)** and November **(B)** 2018. NA denotes genes that lacked an assigned function.

A greater number of statistically significant Bacillariophyta (diatom) gene transcripts were identified in the heated versus control bay sediments at all sampling times (Figure 5 and Supplementary Figure 4). This trend was stronger with more significant Bacillariophyta transcripts in May and June compared with the March and November sampling times. The RNA transcript signature in the heated bay was highly similar in the November and March samplings with transcripts encoding for the genes *COX1-2*, *CYTB*, and *petB*. These genes indicated growth (Rouzer and Marnett, 2009), metabolism (Shan et al., 1990; Rouzer and Marnett, 2009), or other energy demanding activities in the heated bay during winter whereas only one transcript encoding a heat shock gene [*HSP20*; (Liu et al., 2012)] had differentially significant RNA transcripts in the control bay for the November and March samples. Transcripts during the May and June sampling occasions were more diverse when comparing the two bays with the genes *CPS1*, *COX1*, *clpC*, and *LSS*/*ERG7* having higher transcript numbers in the heated bay in May compared to the control bay. These related to various functions including maintaining the cell membrane (Ku et al., 1991), nitrogen homeostasis (Yougo et al., 1991), and energy production (Ku et al., 1991; Corey et al., 1994). In contrast, higher transcripts numbers were identified in the control bay for the genes *CRYAB*, *COX2*, *ALDO*, *RIT2*/*RIN*, and *pps*/*ppsA* that have various functions in e.g., energy production (Rahman et al., 1999) and carbohydrate metabolism (Hutchins et al., 2001; Chiba et al., 2015). Finally, differential Bacillariophyta transcript numbers higher in the heated bay in June included genes related to photosynthesis [*psbV*, *psbC*, and *psaB* (Ikeuchi et al., 1987; Smart and McIntosh, 1991)] and energy production [e.g., *ALDO* and *COX1* (Ku et al., 1991)].

Genes assigned to the Ctenophora only had increased transcript counts in the heated bay in May, June, and November, which included *aprA* (Figure 5 and Supplementary Figure 4) and transcripts encoding *acsB* and *qmoB* were increased in May and November. Furthermore, RNA transcripts assigned to the Spirotrichea were only increased in the heated bay and included *PSAT1* for all sampling points. In May, *PAPSS*, and *EEF1A* also had increased transcript counts.

Dinophyceae (Dinoflagellate) transcripts were generally less common without major differences between the bays (Figure 5 and Supplementary Figure 4). However, there were e.g., higher *EXTL3* gene transcript counts in the heated bay at all sampling times. In contrast, a gene with significantly increased transcript counts in the control bay during May and November encodes histone H1/5 (*H1*_*5*). Pelagophyceae is a subclass of Stramenopiles, commonly called brown algae (Bringloe et al., 2020). Genes with transcripts assigned to the Pelagophyceae had higher transcript counts in both bays including *rfcS* in the control bay and *PAPSS* in the heated bay. Finally, transcripts for Euglenozoa genes also had higher counts in the control bay including *gapA* (glyceraldehyde 3-phosphate dehydrogenase).

## Discussion

This study demonstrated that more than 50 years of heating of a coastal marine system has led to changes in the sediment-bound eukaryotic community structure and activity. Overall, the results showed that long-term warming increased the diversity of the eukaryotic community during spring and triggered changes in the seasonal activity patterns of common sediment eukaryotes. The likely main driver for these changes was temperature with on average 5.1°C higher temperatures in the heated compared to the control bay over an annual cycle (Seidel et al., 2022b). The canonical correspondence analysis separated the eukaryotic communities between the heated and control bay along the salinity differences among sampling sites. Although temperature was not significant, the direction of the temperature and salinity arrows were in the same direction. Other factors such as oxygen concentration is lowered in higher temperatures (Brewer and Peltzer, 2017) such that a lack of oxygen likely limited nitrification in the sediment (Liikanen et al., 2002). This resulted in less nitrite and nitrate, potentially explaining a portion of the observed among-site variation. This study complements and adds a layer of generality to previous studies suggesting significant effects of climate change on the more intensively studied prokaryotic communities (Seidel et al., 2022a,b, 2023).

A highly diversified eukaryote community can act as a reservoir of rare and dormant taxa, which provides ecosystems with a biological buffering capacity to handle climate change and cope with extreme weather events such as marine heat waves (Capo et al., 2016). The Shannon H index was significantly higher in the heated bay in May (Figure 2A). This was potentially explained by an earlier onset of summer conditions in the heated bay, leading to a more diverse active community in the warmer waters in the heated bay compared to the control bay (Salonen et al., 2019). The alpha diversity also varied in November with significantly higher family level alpha diversities in the control bay (Figure 2A). However, the number of statistically increased RNA transcript numbers was still higher in the heated bay (Figures 4, 5). These two opposing patterns may suggest the control bay entered cold winter conditions earlier, which might have caused the community to have a low number of transcripts from a wide range of taxa. While in the heated bay, there were still certain taxa that were more active and dominant while others had a low number of transcripts. In June and March, the active sediment eukaryotic communities in both heated bay and control bay were in summer and spring-bloom conditions. These conditions tended to provide settings for a more similar in alpha diversity. Recent climate change has already altered the marine eukaryotic diversity globally, e.g., in temperate zones and high latitude areas (Gao et al., 2018; Han et al., 2022) and the impacts of climate change can either work directly on the eukaryote’s life-cycle, or indirectly through food web interactions (Przytulska et al., 2015). Eukaryotic diversity in the marine ecosystem generally declines toward the poles, which is suggested to be primarily driven by decreasing of ocean temperatures (Ibarbalz et al., 2019). It is unknown how climate change will affect the sediment-bound eukaryotic diversity, but this study suggested that climate change will alter the community composition, but the magnitude of those changes can vary among different seasons. Other studies have also shown that temporal changes like year and season affect the diversity of sediment eukaryotes (Guardiola et al., 2016; Salonen et al., 2019; Broman et al., 2020; Lalzar et al., 2023). The community composition of sediment eukaryotes also changes in response to annual and seasonal abiotic fluctuations as well as to clines and gradients in environmental variables such as turbidity, nutrients, and human activities (Broman et al., 2015; Salonen et al., 2019; Vause et al., 2019). In all, the data suggested that large scale climate change induced shifts in seasonality that will have major effects on eukaryotic sediment organisms and the overall ecosystem structure and functioning.

Most previous marine sediment bound eukaryote research focuses on spatial variability in community composition (Salonen et al., 2019; Wang et al., 2022; Lalzar et al., 2023) or the temporal variability in the same area (Salonen et al., 2019). This study compared seasonal variability in community composition between two bays in close spatial distance, but with significant differences in water temperatures. This enabled inference of how environmental variation, such as climate change, affects community composition and transcriptomic activity without a significant spatial variance. Furthermore, while most previous studies use 18S rRNA gene data to determine eukaryotic community compositions, this study added the aspect of studying the transcript-based active members. The sediment-bound eukaryote community compositions in this study were dominated by the functionally important Bacillariophyta (diatoms) in both bays, followed by Dinophyceae (dinoflagellates), Ctenophores, and other less abundant taxa. Within these major groups there were both facultative sediment dwellers as well as those organisms that only spend part of their life cycle in the sediment, e.g., as resting stages (Fryxell, 1983; Lewis et al., 1999; McQuoid et al., 2002; Ellegaard and Ribeiro, 2018). This is in line with other studies demonstrating that eukaryotes in sediments have diverse communities with different dominance patterns, e.g., diatoms, maxillopods, or dinoflagellates (Salonen et al., 2019; Iburg et al., 2021; Lalzar et al., 2023). The largest phyla and family level community differences between the bays were present in spring and summer with a tendency toward more diatoms in the control bay (∼75% relative abundance), whereas dinoflagellates contributed more to the relative abundance in the heated bay, albeit not significantly. In contrast, during winter the community compositions between the two bays were more similar. Dinoflagellates are suggested to be favored (Vehmaa et al., 2011) while diatoms have a tendency to decrease (Wasmund et al., 1998) with mild winters. This shift in the relative abundances of diatoms and dinoflagellates is projected to lead to differences in the production of the system (Wei et al., 2004), which might result in lower benthic production and higher pelagic secondary production through energy transfer in the food web (Hjerne et al., 2019). Likewise, differences in the sediment-bound eukaryotic communities in terms of resting stages could lead to an altered benthic-pelagic coupling in coastal systems.

The differential RNA transcript counts showed a similar trend as the alpha diversity, with more increased transcripts in May and November compared to March and June samplings. The most common taxa among those differentially expressed transcripts were the functionally important Bacillariophyta (diatoms) and Dinophyceae (dinoflagellates) that are commonly found in sediments (Broman et al., 2017; Salonen et al., 2019). Bacillariophyta (diatoms) play a vital role in the marine ecosystem (Nelson et al., 1995) and tend to dominate phytoplankton communities in well-mixed coastal areas, where they can access sufficient light and nutrient resources (Morel and Price, 2003). The Bacillariophyta usually bloom in open waters during spring and then sink to the sediment in response to nutrient depletion, but there are also diatoms that solely live on surfaces and in the sediment (Smetacek, 1985; Vyverman, 1992; Dela-Cruz et al., 2006). A previous study found that diatoms dominate a Baltic Sea sediment transcriptome (Broman et al., 2017) with RNA reads associated with diatoms being linked to the thylakoid membrane in the chloroplast and photosynthesis. Furthermore, a low abundance of genes coding for the Calvin-Benson-Bassam cycle (to synthesize Rubisco for photosynthesis) is suggested to indicate that many of these diatoms were in resting stages (Thureborn et al., 2016; Broman et al., 2017). Differential RNA transcripts assigned to diatoms during winter in the heated bay were associated with energy production and protein synthesis, which may indicate an increased metabolism in the heated bay sediment-bound community during winter. In general, the results showed a pattern of early diatom activity in the heated bay suggesting future climate change might decrease the dominance and activity of diatoms in later spring development.

Dinoflagellates were another common component in the sediment eukaryotic community, but only a few genes had differential RNA transcripts that were predominantly in the heated bay. Likewise, the Ctenophores, Spirotrichea, Mamiellophyceae, Zygnemophyceae, and Trebouxiophyceae only had differential transcript numbers in the heated compared to the control bay. These transcripts were associated with various genes and commonly occurring categories included “regulating energy metabolism” and “protein families: metabolism,” which might indicate that the sediment bound eukaryotic community in the heated bay had a more active metabolism throughout the year. The Ctenophores are functionally important gelatinous zooplankton in marine food webs that predate other zooplankton and fish eggs (Gamble, 1994; Mills, 1995; Purcell, 1997). In agreement with this study, they are often abundant during summer phytoplankton blooms and are predicted to be favored by climate change (Mills, 2001; Javidpour et al., 2009). Spirotrichea is a diverse subgroup of the class Ciliophora (commonly termed ciliates) (Gao et al., 2016) that are important components of marine food webs, acting as the food resource for large zooplankton and predators of bacteria and phytoplankton (Weisse and Sonntag, 2016). Other taxa with differential transcripts in the heated bay were Mamiellophyceae, Zygnemophyceae, and Trebouxiophyceae that are phytoplanktonic green algae (Gontcharov et al., 2003; Leliaert et al., 2012). Transcripts from those taxa were mostly involved in photosynthesis and significantly increased in the heated bay especially in May, suggesting the photosynthesizing algal community activities were different between the bays, or due to differences in cell numbers. All these taxa had only differential transcript numbers in the heated bay and their diverse functions suggested that future climate change and warming can trigger more diverse community composition involved in various metabolism pathways.

In summary, this study demonstrated that long-term (> 50 years) heating of coastal waters (average 5°C, i.e., comparable to the expected temperature increase for the Baltic Sea by 2100) altered the community composition, seasonal dynamics, and the transcriptome of sediment-bound eukaryotes. Other studies suggest that sediment eukaryotes show promise to become a tool for environmental monitoring of coastal systems (Gielings et al., 2021). Therefore, the results from this study can help predict the influence of future global warming in sediment communities and their overlaying pelagic systems, with this study suggesting a generally more active metabolism during winter. However, the methodology for characterizing sediment eukaryotes is still under intense development (Reñé et al., 2020), and future work can be extended both spatially and temporally to gain a more comprehensive view of the sediment bound communities.

## Data availability statement

The RNA transcript raw reads are available on the JGI Integrated Microbial Genomes and Microbiomes (IMG) database with the following references JGI proposal ID 503869. The R code used for analysis is available at GitHub: https://github.com/lsjmouse/eukaryotes_manuscript_R_analysis.

## Author contributions

SL: Formal analysis, Methodology, Visualization, Writing – original draft, Writing – review & editing. EN: Formal analysis, Methodology, Software, Supervision, Visualization, Writing – original draft, Writing – review & editing. LS: Data curation, Methodology, Supervision, Writing – original draft, Writing – review & editing. MK: Funding acquisition, Supervision, Writing – original draft, Writing – review & editing. AF: Funding acquisition, Supervision, Writing – original draft, Writing – review & editing. MD: Funding acquisition, Methodology, Supervision, Writing – original draft, Writing – review & editing. SH: Funding acquisition, Methodology, Supervision, Writing – original draft, Writing – review & editing.
