# Supplementary material for: Baltic Sea coastal sediment-bound eukaryotes have increased year-round activities under predicted climate change related warming
Source: Front Microbiol. 2024 Mar 26;15:1369102. doi: 10.3389/fmicb.2024.1369102 (PMC11002985; doi:10.3389/fmicb.2024.1369102)
Supplement: Supplementary file 1 [file Data_Sheet_1.docx]

Supplementary Material

**Baltic Sea Coastal Sediment-Bound Eukaryotes Have Increased
Year-Round Activities Under Predicted Climate Change Related Warming**

Songjun Li, Emelie Nilsson, Laura Seidel, Marcelo Ketzer,
Anders Forsman, Mark Dopson, & Samuel Hylander

**Supplementary Table 1** Details of the sampling, sequencing information, and resulting data used in the study. Data previously published in Seidel et al. (2022) Weakened resilience of benthic microbial communities in the face of climate change. ISME Communications 2: 21.

A separate Excel file has been uploaded.

**Supplemental Table 2** The *p*-values and the log fold changes values for each eukaryotic phylum in each month between the heated and control bays from ANCOM-BC2.

| **Phylum** | ***p*-value May** | ***p*-value June** | ***p*-value November** | ***p*-value March** | **Log fold changes May** | **Log fold changes June** | **Log fold changes November** | **Log fold changes March** |
| --- | --- | --- | --- | --- | --- | --- | --- | --- |
| Stramenopiles | 0.22 | 0.68 | 0.88 | 0.00 | -0.52 | -0.11 | -0.06 | -0.99 |
| Hacrobia | 0.20 | 0.79 | 0.05 | 0.03 | -0.75 | -0.08 | -1.11 | -0.90 |
| Amoebozoa | 0.50 | 0.72 | 0.16 | 0.08 | -0.27 | -0.18 | -0.81 | -0.82 |
| Alveolata | 0.48 | 0.20 | 0.16 | 0.08 | 0.48 | 0.37 | -0.49 | -0.72 |
| Opisthokonta | 0.34 | 0.65 | 0.76 | 0.01 | 0.63 | 0.19 | 0.21 | -0.88 |
| Archaeplastida | 0.78 | 0.83 | 0.01 | 0.01 | -0.15 | -0.05 | -0.83 | -1.01 |
| Rhizaria | 0.12 | 0.33 | 0.45 | 0.26 | 0.96 | 0.39 | -0.97 | -0.71 |
| Apusozoa | 0.06 | 0.10 | 0.00 | 0.08 | 1.26 | 0.67 | -2.83 | -1.11 |
| Excavata | 0.30 | 0.75 | 0.32 | 0.00 | 0.47 | -0.10 | -0.50 | -1.15 |
| NA uncl. | 0.81 | 0.54 | 0.20 | 0.08 | -0.16 | 0.21 | -0.46 | -0.81 |

**Supplemental Table 3** Variance inflation factor (VIF) for CCA to test if environmental variables add explanatory information to the differences between communities. VIF < 5 indicate variables still explain part of the differences between bays communities. Here VIF of pH and Sulfate were >5. Therefore, they were excluded from following CCA and following permutation test.

| Environmental variable | VIF |
| --- | --- |
| Total Iron | 3.930336 |
| pH | 5.018321 |
| Nitrate | 2.786679 |
| Nitrite | 4.507576 |
| Ferrous Iron | 3.543763 |
| Temperature | 1.970521 |
| Oxygen | 2.114424 |
| Organic matter | 2.095945 |
| Depth | 3.753154 |
| Sulfate | 5.463654 |
| Salinity | 1.468183 |
| Phosphate | 3.443024 |

**Supplemental Table 4** Permutation ANOVA test for CCA under direct model with marginal effects of terms within each bay. Number of permutations n= 999. Test of which environmental variables significantly explain variation on microbial communities in each bay.

| Variables | Df | ChiSquare | F | p-value |
| --- | --- | --- | --- | --- |
| Total Iron | 1 | 0.20652 | 1.0055 | 0.343 |
| Nitrate | 1 | 0.14462 | 0.7041 | 0.969 |
| Nitrite | 1 | 0.29098 | 1.4167 | 0.07 |
| Ferrous Iron | 1 | 0.18196 | 0.8859 | 0.869 |
| Temperature | 1 | 0.26836 | 1.3066 | 0.188 |
| Oxygen | 1 | 0.1863 | 0.9071 | 0.643 |
| Organic matter | 1 | 0.21364 | 1.0402 | 0.288 |
| Depth | 1 | 0.2722 | 1.3253 | 0.02* |
| Salinity | 1 | 0.34096 | 1.6601 | 0.029* |
| Phosphate | 1 | 0.26041 | 1.2679 | 0.08 |

***: p<0.001 **: p<0.01 *: p<0.05

**Supplementary Figure 1** Relative abundance of RNA transcripts at the level of domain as well as viruses.

**
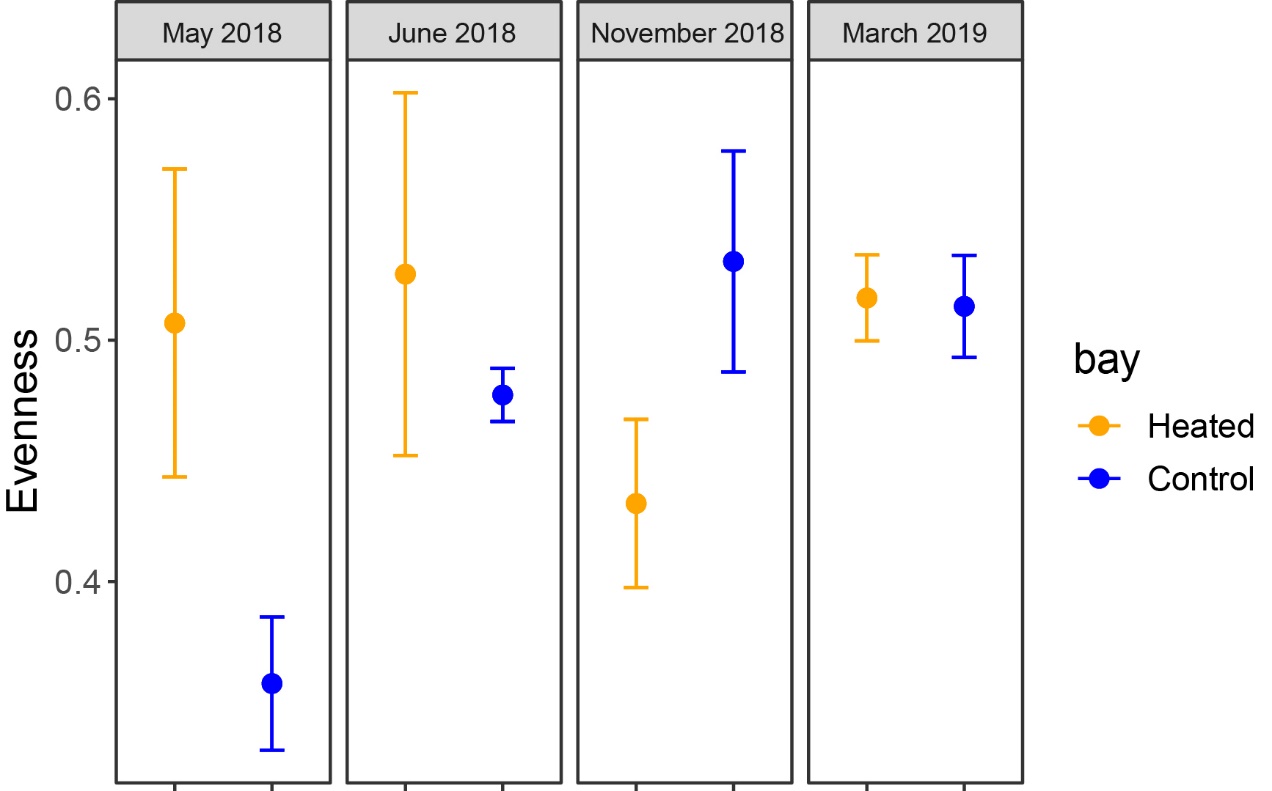
**

**Supplementary Figure 2** Shannon ́s evenness at the level of family for the heated (orange) and control (blue) bays for each sampling month.

**Supplementary Figure 3** Mean-Difference (MD) plot of transcript counts between the heated and control bays for the combined data for all sampling times. Genes with fold-changes significantly greater than 1.5 are highlighted for the heated (orange) and control (blue) bays for each sampling month.


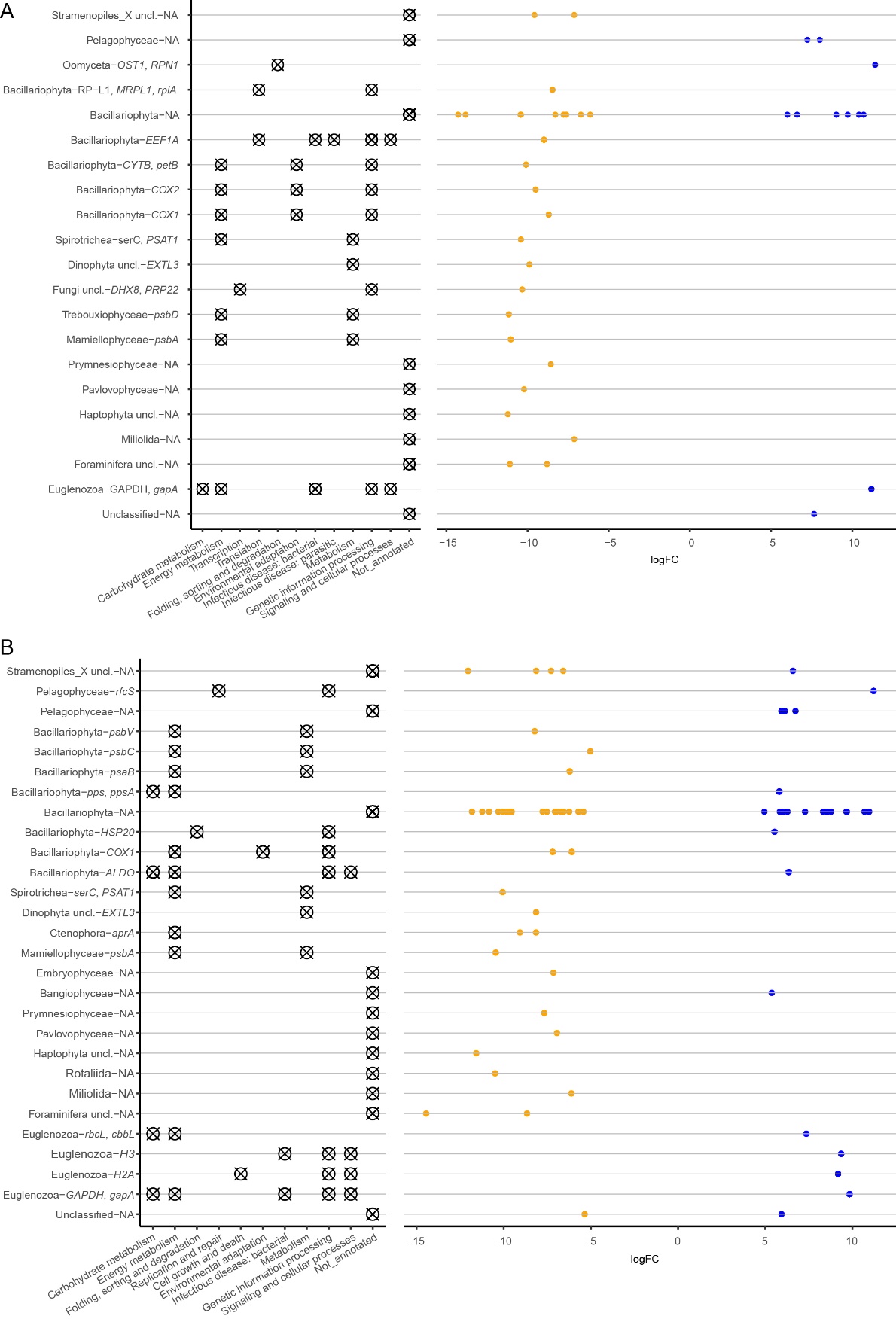


**Supplementary Figure 4** Taxonomy and metabolic functional groups of differential RNA transcript numbers (logFC) for genes between heated bay and control bay in March 2019 (A) and June 2018 (B). NA denotes genes that lacked an assigned function.


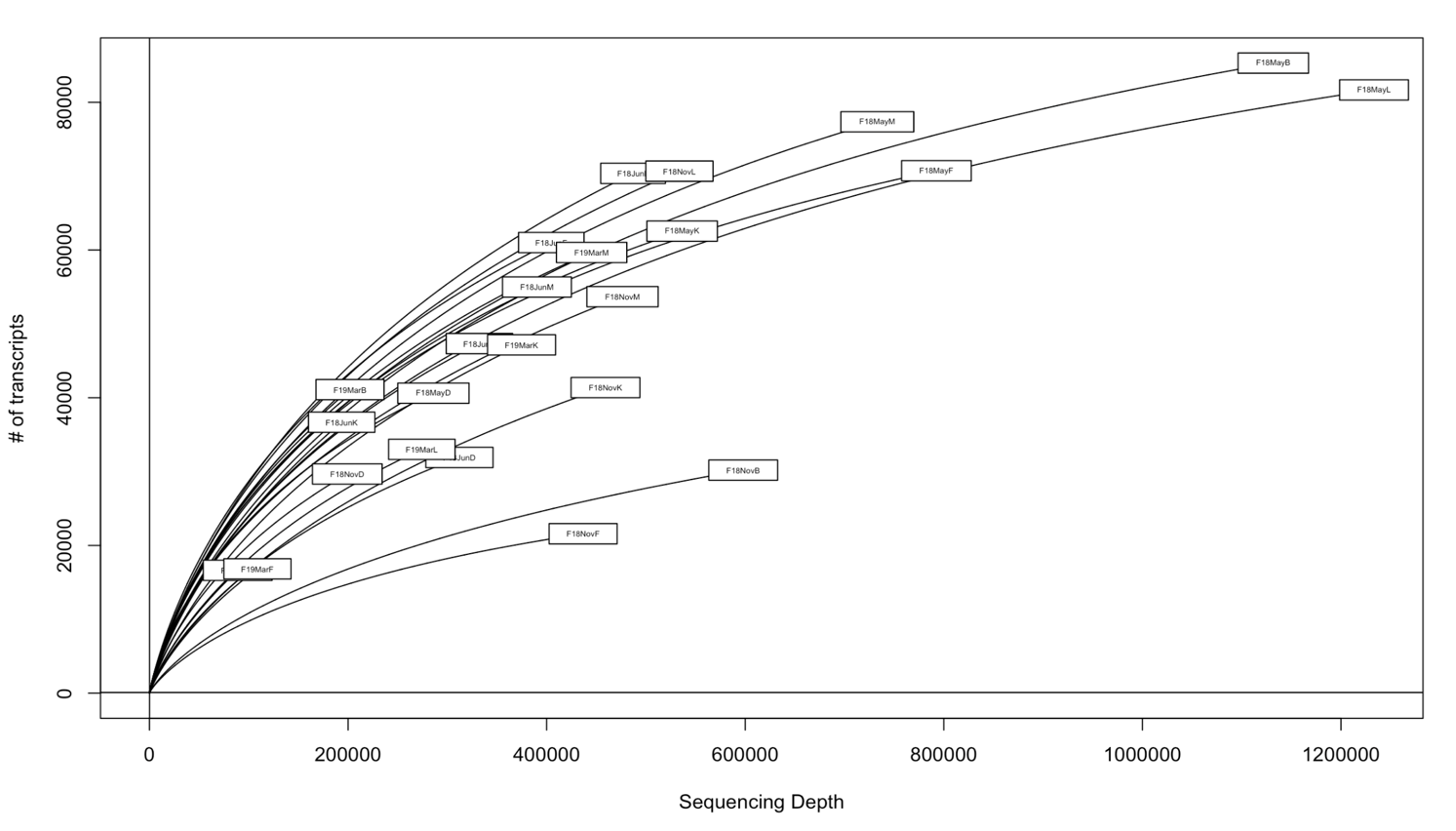


**Supplementary Figure 5** Rarefaction curve figure on the filtered eukaryotic RNA reads.
